# Immune checkpoint molecules B7-H6 and PD-L1 co-pattern the tumor inflammatory microenvironment in human breast cancer

**DOI:** 10.1038/s41598-021-87216-9

**Published:** 2021-04-06

**Authors:** Boutheina Cherif, Hana Triki, Slim Charfi, Lobna Bouzidi, Wala Ben Kridis, Afef Khanfir, Kais Chaabane, Tahya Sellami-Boudawara, Ahmed Rebai

**Affiliations:** 1 Laboratory of Molecular and Cellular Screening Processes, Center of Biotechnology of Sfax, Sfax University, B.P 1177, 3018 Sfax, Tunisia; 2 Department of Pathology, Habib Bourguiba University Hospital, Sfax, Tunisia; 3 Department of Medical Oncology, Habib Bourguiba University Hospital, Sfax, Tunisia; 4 Department of Gynecology, HédiChaker University Hospital, Sfax, Tunisia

**Keywords:** Breast cancer, Cancer microenvironment, Tumour immunology, Cell biology, Immunology

## Abstract

B7-H6 and PD-L1 belong to the B7 family co-stimulatory molecules fine-tuning the immune response. The present work investigates the clinical effect of B7-H6 protein expression with PD-L1 status and the infiltration of natural killer cells as potential biomarkers in breast tumor inflammatory microenvironment. The expression levels of B7-H6 protein by cancer cells and immune infiltrating cells in human breast cancer tissues and evaluate their associations with PD-L1 expression, NK cell status, clinical pathological features and prognosis were explored. The immunohistochemistry labeling method was used to assess B7-H6 and PD-L1 proteins expression by cancer and immune cells. The associations between immune checkpoint, major clinical pathological variables and survival rates were analyzed. B7-H6 protein was depicted in both breast and immune cells. Results showed that Tumor B7-H6 expression is highly associated with Her-2 over expression. B7-H6 + immune cells are highly related to the Scarff–Bloom–Richardson grade and associated with PD-L1 expression and NK cells status. Survival analysis revealed a better prognosis in patients with low expression of B7-H6 by cancer cells. Conversely, B7-H6 + immune cells were significantly associated with longer survival. Findings strongly suggest an interaction between B7 molecules that contributes to a particular design of the inflammatory microenvironment. This may influence the efficiency of therapies based on antibodies blocking the PD-L1/PD1 pathway and can explain the detection of clinical benefits only in a fraction of patients treated with immune checkpoint inhibitors.

## Introduction

Breast cancer is the most diagnosed malignancy in women worldwide and is a highly heterogeneous disease presenting a broad range of molecular and clinical characteristics^1^. Towards this heterogeneity, the establishment of an effective immune response requires the participation of several actors. Both adaptive and innate immunity contribute to the immune editing of malignancy features^2,3^. However, more attention has been devoted to natural immunity. Natural killer (NK) cells are important components of the innate immune system and play a central role in the tumor microenvironment (TME) design^4^ since they promote the adaptive response through their cytokine secretion polarizing T-cell activation^5–7^.

Moreover, the modulation of NK cells response can be controlled by the immune checkpoint molecules of the B7 family^8^. The most prominent immune checkpoint regulators are programmed cell death 1 (PD-1)/PD-1 ligand 1 (PD-L1) and CTL antigen 4 (CTLA-4). These are highlighted in a variety of cancers and their therapeutic advances have encouraged researchers to investigate other targets from the B7/CD28 family. Recently, five new B7 family ligands, B7-H3, B7-H4, B7-H5, B7-H6, and B7-H7, were identified and increasingly investigated in various solid cancers to seek significant association with cancer progression and patient prognosis in different clinical retrospective studies^9–12^.

Among this group, B7-H6 seems to be a potential target for a new immunotherapy strategy.B7-H6 (also known as NCR3LG1) is a ligand for the NK-cell–activating receptor NKp30^13^. Human B7-H6 protein is rarely expressed in normal tissues, but is overexpressedin various primary human tumors, including leukemia, lymphoma, and gastrointestinal stromal tumors^14^. However, B7-H6 can be induced at the surface of CD14(+)/CD16(+) proinflammatory monocytes and neutrophils upon stimulation by ligands of Toll-like receptors or proinflammatory cytokines such as IL-1β and TNF-α^15^. B7-H6 binds to NKp30 through the complementarity-determining region (CDR)-like loops of its V-like domain in an antibody-like interaction^16^. NK cells eliminate tumor cells that express B7-H6 directly by cytotoxicity through co-signals balancing between activatorsand inhibitors, or indirectly by cytokines release. Hence, the expression of B7-H6 by tumor cells is an important mechanism involved in the activation of innate immunity mediated by NK cells. However, to prevent NK-mediated recognition and thus divert this visibility to the immune system, the malignant cells release or hide the B7-H6 molecule^17^.

The clinical significance of B7-H6 expression in human cancers has little been investigated. In ovarian cancer, positive B7-H6 staining was predominantly observed on the membrane and in the cytoplasm of the malignant cells. The overall survival rate of the subgroup with lower B7-H6 expression was significantly better compared to those with higher B7-H6 expression^18^. In astrocytoma, B7-H6 positive expression was significantly associated with World Health Organization Grade^19^. Recently, high expression of B7-H6 was considered as a predictor of poor prognosis in esophageal squamous cells^20^. In the case of breast cancer disease, B7-H6 expression was revealed to be an unfavorable prognosis biomarker^21^.

However, these data showed the expression of B7 molecules severally and the association between B7-H6 and PD-L1 expression in human cancer remains unknown. In view of the significance of the B7 molecules in the immune surveillance, the aim of the current study is to investigate the co-expression profiles and the clinical significance of B7-H6 and PD-L1 in women breast cancer. We assessed the contribution of B7-H6 and PD-L1 expression by tumor cells and immune cells infiltrating the tumor microenvironment. We also examined and compared their relationship with NK cells status in controlling patient’s survival.

## Results

### B7-H6 and PD-L1 immunodetection in breast cancer

**Figure 1:**
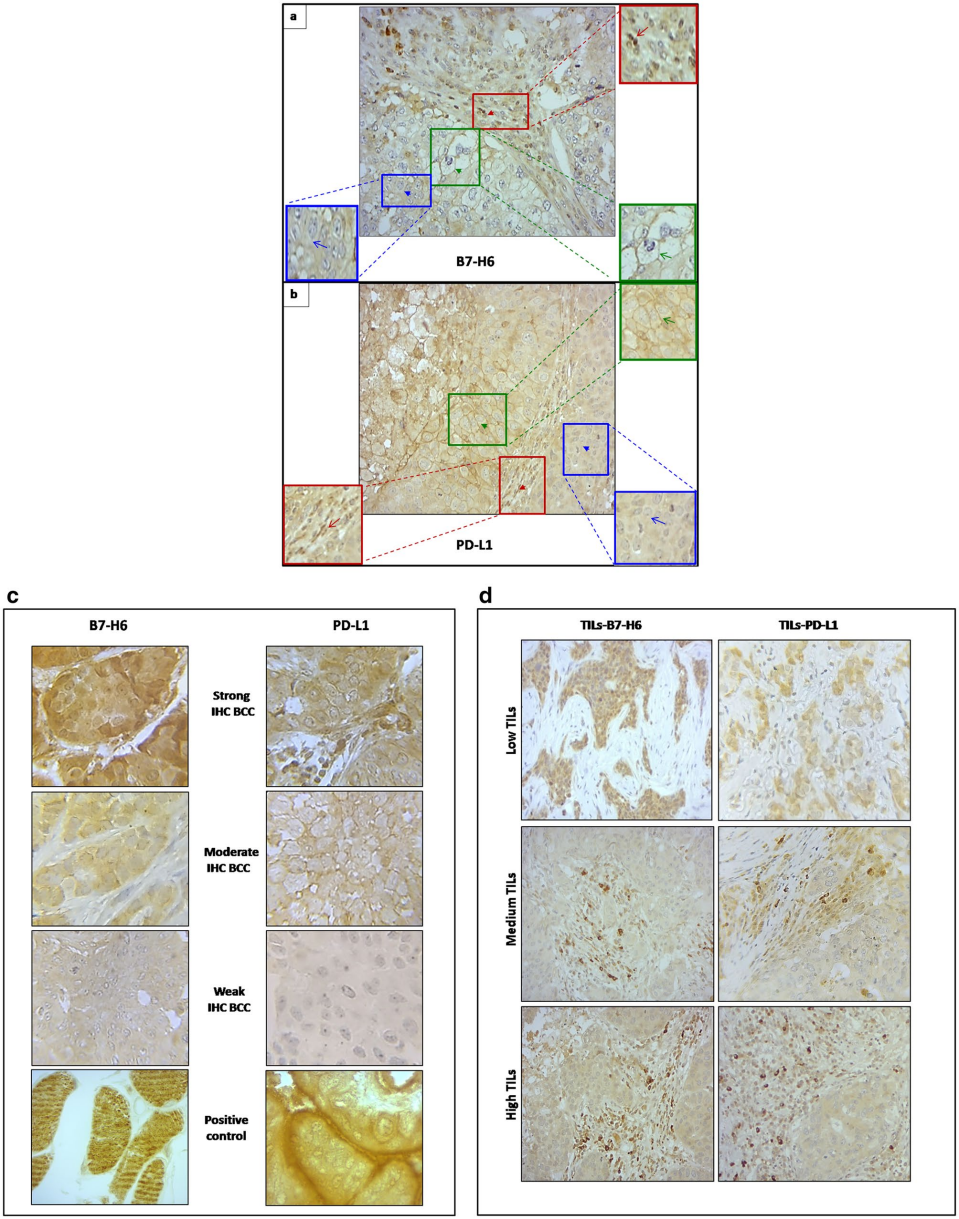
Representative images of immune checkpoint molecules B7-H6 and PD-L1 localizations and abundance in breast cancer tissues using immunohistochemical staining. B7-H6 IHC analysis showed three localizations (**a,** Original magnification × 400): membranous tumor cells (**a**, green gated area), cytoplasmic tumor cells (**a**, blue gated area) and in the stroma area stained on immune infiltrated cells (TILs) (**a**, red gated area). Identically, PD-L1 IHC analysis showed three localizations (**b,** Original magnification × 400): membranous tumor cells (**b**, green gated area), cytoplasmic tumor cells (**b**, blue gated area) and in the stroma on TILs (**b**, red gated area). **(c,** Original magnification × 1000**)** B7-H6 and PD-L1 expression analysis by immunohistochemical staining at the level of breast cancer cells (IHC BCC). B7-H6 and PD-L1 IHC showed positive stained tumor cells at three different intensities; strong, moderate and weak. Positive controls for IHC staining of B7-H6 (Striated muscles tissue) and PD-L1 (Placenta tissue) respectively. (**d**) Original magnification × 400**)** B7-H6 and PD-L1 IHC showed positive stained immune cells in three different densities; Low, Medium and High B7-H6 + TILs or PD-L1 + TILs distribution in the stroma area are presented.

The immunohistochemistry analysis showed positive staining for B7-H6 (Fig. 1a) and PD-L1 (Fig. 1b) both on the membrane and in the cytoplasm of cancer cells, but also in TILs (Fig. 1a,b). Representative examples of B7-H6 and PD-L1immunostaining in breast cancer cells (BCC) and TILs are shown in Fig. 1c,d respectively. B7-H6 and PD-L1 expressions were evaluated successfully by IHC in 156 BC tissues and positive controls at different sites and intensity (Fig. 1c). Based on the area and intensity of cyto-membranous immunostaining in BCC, H-scores were generated as described in methods. In order to investigate the correlation between clinical parameters and the B7-H6 and PD-L1protein expression levels in BCC, we categorized the 156 patients into two major subgroups according to the intensity of B7-H6 and PD-L1immunohistochemical staining. Data showed that B7-H6 BCC expression levels were low in 61% (0 ≤ H-score < 100) and high in 39% of cases (H-score ≥ 100). For PD-L1BCC distribution in our cohort, our results show that PD-L1BCC expression levels were low in 80% (0 ≤ H-score < 100) and high in 20% of cases (H-score ≥ 100) (Table 1). Regarding TILs density distributions, TILs-B7-H6 + were predominantly localized in the stroma surrounding BCC clusters and abundant in 14% of cases. Conversely, TILs-PD-L1 + were abundant in 60% of cases (Table 1).

**Table 1:** Associations of B7-H6 and PD-L1 expression with clinical parameters in primary breast cancer patients.

Clinical pathological parameter	All patients n = 156	B7-H6 BCC		*p* value	TILs-B7-H6			*p* value	PD-L1 BCC		*p* value	TILs-PD-L1			*p* value
		< median n = 96 (61%)	≥ median n = 60 (39%)		Low n = 126 (86.4%)	High n = 20 (13.6%)	Unk n = 10		< 100 n = 125 (80%)	≥ 100 n = 31 (20%)		Low n = 58 (40%)	High n = 88 (60%)	Unk n = 10	
**Age in years**				1				0.224			0.62				0.461
< median = 48	70	43	27		54	13	3		54	16		23	44	3	
≥ median = 48	86	53	33		72	7	7		71	15		35	44	7	
**Histological type**				0.70				0.426			0.035*				0.26
IDC	129	78	51		102	18	9		99	30		45	75	9	
Others	27	18	9		24	2	1		26	1		13	13	1	
**ER**				0.24				0.002**			0.23				0.09
Negative	57	39	18		39	14	4		42	15		16	37	4	
Positive	99	57	42		87	6	6		83	16		42	51	6	
**PR**				0.98				0.199			0.37				0.68
Negative	69	43	26		52	12	5		53	16		24	40	5	
Positive	87	53	34		74	8	5		72	15		34	48	5	
**Her2**				0.002**				0.787			1				0.31
Negative	106	74	32		87	12	7		84	22		43	56	7	
Positive	50	22	28		39	8	3		41	9		15	32	3	
**Molecular subtypes**				0.003**				0.141			0.30				0.25
H	22	9	13		16	5	1		18	4		6	15	1	
LA	31	23	8		27	1	3		28	3		16	12	3	
LB	28	13	15		23	3	2		23	5		10	16	2	
LB-LIKE	47	27	20		41	5	1		37	10		18	28	1	
TNBC	28	24	4		19	6	3		19	9		8	17	3	
**SBR grading**				0.14				0.0003***			< 0.001***				0.049*
I	23	18	5		21	0	2		23	0		13	8	2	
II	66	41	25		57	4	5		59	7		27	34	5	
III	67	37	30		48	16	3		43	24		18	46	3	
**Tumor size (cm)**				0.84				0.43			0.41				0.91
T1 ≤ 2	29	16	13		22	4	3		26	3		8	18	3	
2 < T2 ≤ 5	87	55	32		72	10	5		65	22		34	48	5	
T3 > 5	21	14	7		14	5	2		18	3		7	12	2	
T4	18	11	7		17	1	0		15	3		8	10	0	
Unknown	1	0	1		1	0	0		1	0		1	0	0	
**Lymphe Nodestatus**				0.43				0.012*			0.57				0.82
N0	56	39	17		43	10	3		42	14		20	33	3	
N1	53	30	23		47	2	4		44	9		21	28	4	
N2	26	16	10		17	7	2		22	4		8	16	2	
N3	19	10	9		17	1	1		15	4		7	11	1	
Unknown	2	1	1		2	0	0		2	0		2	0	0	
**Metastasis**				0.68				0.19			0.43				0.44
M0	108	69	39		83	18	7		86	22		39	62	7	
M +	13	7	6		13	0	0		23	0		7	6	0	
Unknown	35	20	15		30	2	3		16	19		12	20	3	

Variable between groups presented in frequency tables evaluated by Chi-Square test. Two sided *p* values are considered statistically significant if < 0.05 and are indicated in bold.

### Correlation of B7-H6 and PD-L1 expressions with clinical pathological features

The correlations between clinical parameters and B7-H6/PD-L1 expression in patients are reported in Table 1. An overexpression of B7-H6 by cancer cells in Her-2 positive molecular groups was noticed. Statistical analyses indicate a significant correlation of B7-H6 BCC expression with only Her-2 expression (*p* < 0.01) and molecular subtypes (*p* < 0.01). However, we did not find any correlation with SBR grade, tumor size, lymph node invasion or metastasis. For correlation of TILs-B7-H6 status with clinical pathological parameters, we found a significant relation with lymph node status (*p* < 0.05), ER status (*p* < 0.01) and SBR grading (*p* < 0.001). Regarding PD-L1expression, our results display a significant correlation of PD-L1BCC expression with histological type (*p* < 0.05) and SBR grade (*p* < 0.001), but no correlation between clinical parameters and TILs-PD-L1 status was found in patients except for a limited significant correlation with SBR grade (*p* = 0.049).

### Correlations of B7-H6 expressions withPD-L1and NK-TILs status

**Figure 2:**
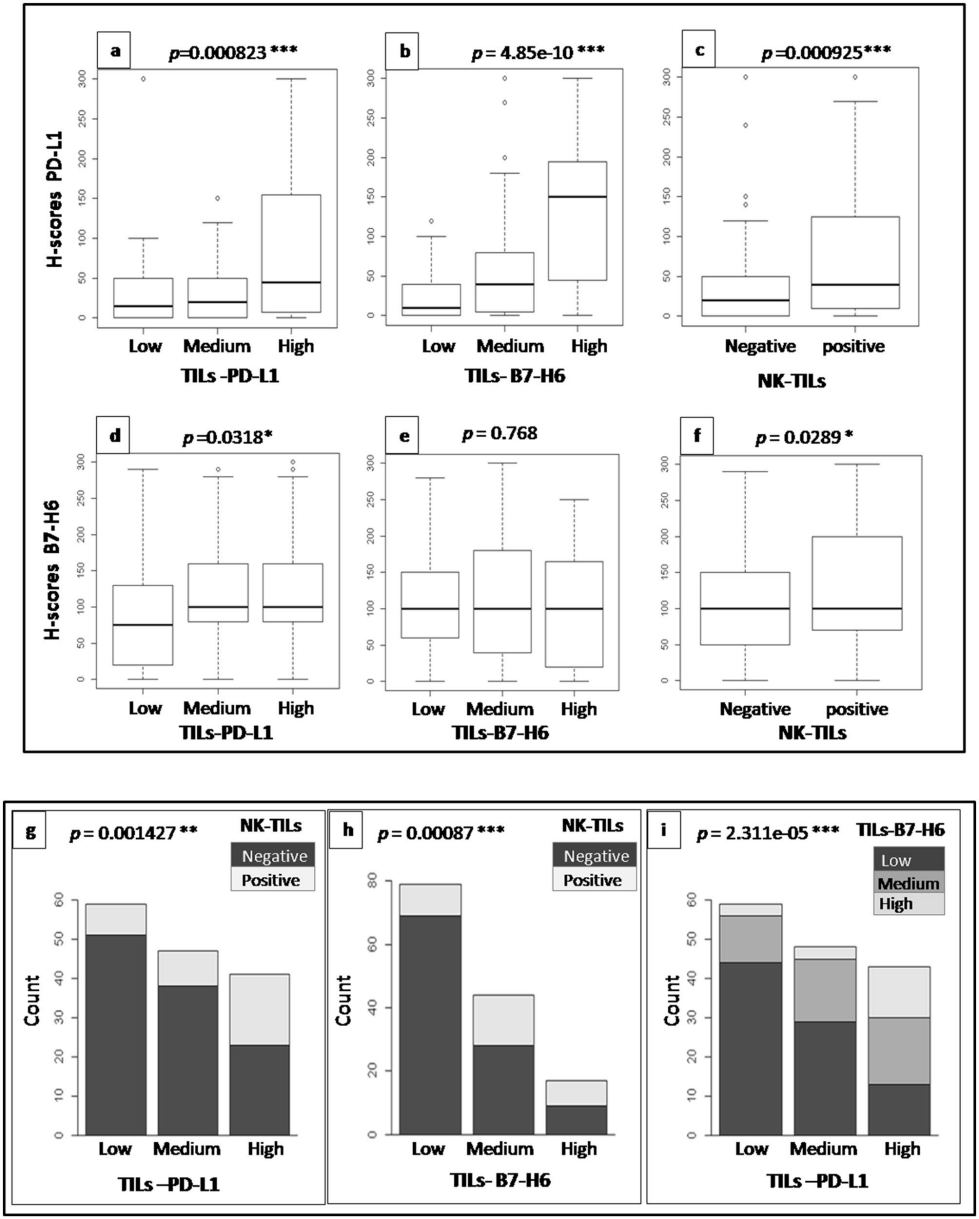
B7-H6 and PD-L1 expressions features associations with TILs-PD-L1, TILs-B7-H6 and NK-TILs status. TILs-B7-H6 and TILs-PD-L1 were evaluated using the three-tier scoring system from high-power field (HPF) areas: 0–5%/HPF (Low), 5–50%/HPF (Medium), > 50%/HPF (High). Boxplot representations of H-score PD-L1 distribution according to TILs-PD-L1 status (**a**, *p* < 0.001), TILs-B7-H6 status (**b**, *p* < 0.001) and NK-TILs status (**c**, *p* < 0.001). Boxplot representations of H-score B7-H6 distribution according to TILs-PD-L1 status (**d**, *p* < 0.05), TILs-B7-H6 status (**e**, *p* = 0.768) and NK-TILs status (**f**, *p* < 0.05). Barplot representations showing NK-TILs distribution according to TILs-PD-L1 status (**g**, *p* < 0.01) and TILs-B7-H6 status (**h**, *p* < 0.001). Barplot representation of TILs-B7-H6 distribution among TILs-PD-L1 status (**I**, *p* < 0.001).

The role of B7-H6 in the inflammatory tumor microenvironment design was sought via the relationship of its expression with PD-L1 and NK-TILs status. For more insight, we conducted two types of analysis. The first evaluated the relations on the raw results either in H-scores for the BCC expression or in TILs scores shared in 3 classes, as described in the materials and methods section. Data of these analyses are shown in Fig. 2. The second way evaluates the relationships between the different parameters after the classification into two classes according to the cut-off mentioned in the materials and methods section and based on previous works^18,22^. Analyses based on raw data show significant correlations of PD-L1BCC expression with the status of NK-TILs (*p* < 0.001), TILs-B7-H6 (*p* < 0.001), and TILs-PD-L1 (*p* < 0.001) (Fig. 2a–c). In contrast, a poor significant correlation of B7-H6 BCC expression with the status of TILs-PD-L1 (Fig. 2d, *p* = 0.0318) and NK-TILs (Fig. 2f, *p* = 0.0289) and an insignificant correlation with TILs-B7-H6 (Fig. 2e) were found. Nevertheless, the TILs-B7-H6 status is strongly correlated with the TILs-PD-L1 status (Fig. 2i, *p* < 0.001) and NK-TILs (Fig. 2h, *p* < 0.001), but TILs-PD-L1 status had a significant lower relationship with NK-TILs infiltration (Fig. 2g, *p* < 0.01). The second way of analysis based on binary classes did not display any significant correlations between B7-H6-BCC expression and all other biomarkers statuses. However, the status of B7-H6-TILs shows significant associations with the status of NK-TILs (*p* = 0.0419), TILs-PD-L1 (*p* = 0.0268) and PD-L1BCC expression (*p* < 0.01). In addition, analysis deciphered a limited significant correlation between the status of NK-TILs and TILs-PD-L1 (*p* = 0.0284) and PD-L1BCC expression (*p* = 0.0232) (Supplementary Table).

### Prognostic significance of B7-H6 expression

**Figure 3:**
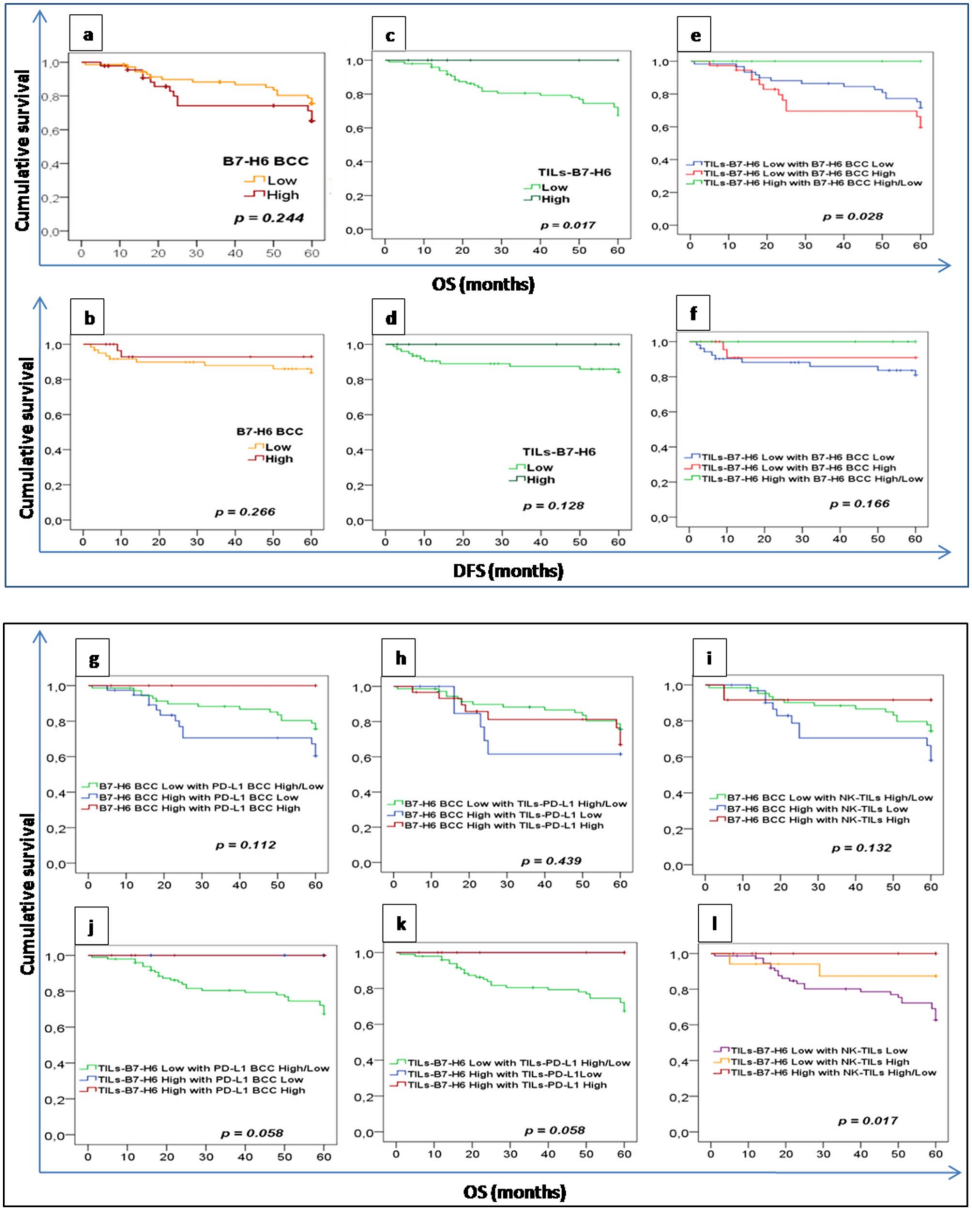
Survival analysis according to B7-H6 expression and subgroups of combination between B7-H6 BCC or TILs-B7-H6 expression and immune parameters in breast cancer tissues. (**a**–**b**) Kaplan–Meier curves stratified on B7-H6 expression by cancer cells (B7-H6 BCC) for overall survival (OS) and disease free survival (DFS). (**c**–**d**) Kaplan–Meier curves stratified on B7-H6 expression by immune infiltrating cells (TILs-B7-H6) for OS and DFS. OS (**e**) and DFS (**f**) curves according to B7-H6 expression by tumor and immune cells together leading to three subgroups: subgroup 1: TILs-B7-H6^Low^/B7-H6 BCC^Low^, subgroup 2: TILs-B7-H6^Low^/B7-H6 BCC^High^ and subgroup 3: TILs-B7-H6^High^/B7-H6 BCC^High/Low^. (**g**) OS curves according to B7-H6 BCC expression combined with PD-L1 BCC expression leading to three subgroups as follows: subgroup 1: B7-H6 BCC^Low^/PD-L1 BCC^High/Low^, subgroup 2: B7-H6 BCC^High^/PD-L1 BCC^Low^ , subgroup 3: B7-H6 BCC^High^/PD-L1 BCC^High^. (**h**) OS curves according to B7-H6 BCC expression combined with TILs-PD-L1 status leading to three subgroups as follows: subgroup 1: B7-H6 BCC^Low^/TILs-PD-L1^High/Low^, subgroup 2: B7-H6 BCC^High^/TILs-PD-L1^Low^, subgroup 3: B7-H6 BCC^High^/TILs-PD-L1^High^. (**i**) OS curves according to B7-H6 BCC expression combined with NK-TILs status leading to three subgroups as follows: subgroup 1: B7-H6 BCC^Low^ NK-TILs^High/Low^, subgroup 2: B7-H6 BCC^High^/ NK-TILs^Low^ ,subgroup 3: B7-H6 BCC^High^/ NK-TILs^High^. (**j**) OS curves according to TILs-B7-H6 status combined with PD-L1 BCC expression leading to three subgroups as follows: subgroup 1: TILs-B7-H6^Low^/ PD-L1 BCC^High/Low^, subgroup 2: TILs-B7-H6^High^/ PD-L1 BCC^Low^, subgroup 3: TILs-B7-H6^High^/ PD-L1 BCC^High^. (**k**) OS curves according to TILs-B7-H6 status combined with TILs-PD-L1 status leading to three subgroups as follows: subgroup 1: TILs-B7-H6^Low^/ TILs-PD-L1^High/Low^, subgroup 2: TILs-B7-H6^High^/TILs-PD-L1^Low^, subgroup 3: TILs-B7-H6^High^/ TILs-PD-L1^High^. (**l**) OS curves according to TILs-B7-H6 status combined with NK-TILs status leading to three subgroups as follows: subgroup 1: TILs-B7-H6^Low^ /NK-TILs^Low^, subgroup 2: TILs-B7-H6^Low^/NK-TILs^High^, subgroup 3: TILs-B7-H6^High^/ NK-TILs^High/Low^.

In order to look for the prognostic value of B7-H6 expression in women breast cancer, the log-rank survival analyses were performed according to B7-H6 expression levels in BCC or TILs scores after classification and collection of survival data. As shown in Fig. 3a, patients with low B7-H6 BCC expression had a markedly longer OS compared to patients with high B7-H6 expression, however, DFS curves are inverted but almost confused (Fig. 3b). In regards to TILs-B7-H6 status in survival analysis (Fig. 3c,d), both OS and DFS rates of the subgroup with higher TILs-B7-H6 + are longer than those of the subgroup with lower TILs-B7-H6 + , Kaplan–Meier survival curves for OS show significant differences between the two groups (*p* = 0.017) but no significance with DFS curves (*p* = 0.128). We then addressed the prognostic relevance of B7-H6 expression by tumor cells or TILs, we investigated survival data among combined groups; group 1 = TILs-B7-H6^Low^/B7-H6 BCC^Low^, group 2 = TILs-B7-H6^Low^/B7-H6 BCC^High^ and group 3 = TILs-B7-H6^High^ /B7-H6 BCC^Low/High^. As shown in Fig. 3 patients from the third group had better survival rates compared to patients belonging the others two groups with significantly longer OS (Fig. 3e, *p* = 0.028) but insignificant longer DFS (Fig. 3f, *p* = 0.166).

### Prognostic significance of the concomitant expression of B7-H6 and the others immune inflammatory biomarkers

To better understand the impact of B7-H6 expression in relation with PD-L1 and NK cell status on patient’s survival, different subgroups were created taking into account the classifications according to PD-L1 and NK-cell statues. Figure 3g–i show survival curves (OS only) of subgroups generated by combinations between B7-H6 BCC status with PD-L1 BCC or TILs-PD-L1or NK-TILs status. Figure 3j–l show survival curves (OS) of subgroups generated by combinations between TILs-B7-H6 status and PD-L1 BCC or TILs-PD-L1 or NK-TILs status. Regardless of the three types of combinations, patients with B7-H6 BCC^High^/PD-L1 BCC^Low^ (Fig. 3g) or B7-H6 BCC^High^ /TILs-PD-L1^Low^ (Fig. 3h) or B7-H6 BCC^High^ /NK-TILs^Low^ (Fig. 3i) expression status have obviously the shorter OS among the others subgroups. Conversely, OS curves of TILs-B7-H6 combination groups show that a longer survival is always observed in the subgroup with the high profile of TILs-B7-H6 associated with high status of either PD-L1 BCC (Fig. 3j) or TILs-PD-L1 (Fig. 3k) or NK-TILs (Fig. 3l). The differences are important in the case of overall survival to achieve significant variations in the combinations TILs-B7-H6^High^/NK-TILs^High^ (*p* = 0.017). Nevertheless, TILs-B7-H6^High^ group in combination with PD-L1BCC or TILs-PD-L1 groups always shows a better survival (*p* = 0.058). In regards to multivariate analyses, cox-regression showed a significant effect of TILs-B7-H6 (hazard ratio [HR] = 0.038, 95% confidence interval (CI) = 0.01–0.730, *p* = 0.018), where all 20 patients with high expression have survived. Furthermore, association of NK-TILs status with OS is also significant in multivariate analyses (Cox-regression) after adjusting for Her2 status (HR = 0.169, CI = 0.039–0.728, *p* = 0.017).

### Prognostic significance of B7-H6 besides others immune inflammatory biomarkers in Her2 positive cancer samples

To better understand the impact of B7-H6 and others immune checkpoint molecules within Her2 positive cancer subtype, we sought correlations between clinical parameters and B7-H6 or PD-L1 expressions by tumor cells and TILs (Table 2), statistical analysis show only significant associations of B7-H6 or PD-L1 BCC expression with SBR grade (*p* = 0.025 and *p* = 0.035 respectively) but a limited significant association between TILs-B7-H6 status and lymph node invasion (*p* = 0.055). Furthermore associations between immunological parameters were performed (Supplementary Fig. 1), results show the same trends across the whole cohort with the exception of the relationship between B7-H6 BCC expression and TILs-B7-H6 status where boxplots show a limited significant association between them (Supplementary Fig. 1e, *p* = 0.051). Similarly, but with less intensity in *p*-value, data indicate significant correlations between PD-L1 BCC expression and the status of TILs (Supplementary Fig. 1a–c, *p* < 0.05). Regarding the prognostic value of B7-H6 and PD-L1 through survival analyzes among Her2 positive patients, the same observations were recorded with OS curves in whole cohort analyzes showing longer survival for patients with high TILs-B7-H6 status and shorter survival for patients with high B7-H6 BCC expression (supplementary Fig. 2a–c). In contrast, no difference in OS curves was observed in high or low TILs-PD-L1 presence but patients with high PD-L1 BCC expression tended to have a better survival (supplementary Fig. 2d–f).

**Table 2:** Associations of B7-H6 and PD-L1 expression with clinical parameters in Her2 positive primary breast cancer patients.

Clinical pathological parameter	All patients n = 50	B7-H6 BCC		*p* value	TILs-B7-H6			*p* value	PD-L1 BCC		*p* value	TILs-PD-L1			*p* value
		< median n = 22	≥ median n = 28		Low n = 39	High n = 8	UnK n = 3		< median n = 41	≥ median n = 9		Low n = 15	High n = 32	Unk n = 3	
**Age in years**				0.77				0.75			0.11				0.38
< median = 48	27	11	16		22	5	0		20	7		10	17	0	
≥ median = 48	23	11	12		17	3	3		21	2		5	15	3	
**Histological type**				1				0.34			0.26				0.75
IDC	45	20	25		35	8	2		36	9		14	29	2	
Others	5	2	3		4	0	1		5	0		1	3	1	
**ER**				0.61				0.11			0.26				0.68
Negative	25	11	14		18	6	1		19	6		7	17	1	
Positive	25	11	14		21	2	2		22	3		8	15	2	
**PR**				0.77				0.85			0.76				0.96
Negative	30	14	16		23	5	2		25	5		9	19	2	
Positive	20	8	12		16	3	1		16	4		6	13	1	
**Molecular Subtypes**				0.94				0.32			0.91				0.52
H	22	10	12		17	5	0		18	4		6	16	0	
LB	28	12	16		22	3	3		23	5		9	16	3	
**SBR grading**				0.025*				0.10			0.035*				0.75
I	3	3	0		2	0	1		3	0		1	1	1	
II	22	12	10		19	1	2		21	1		7	13	2	
III	25	7	18		18	7	0		17	8		7	18	0	
**Tumor size (cm)**				0.92				0.80			0.43				0.13
T1 ≤ 2	8	3	5		5	2	1		7	1		0	7	1	
2 < T2 ≤ 5	24	11	13		19	4	1		18	6		9	14	1	
T3 > 5	8	3	5		7	1	0		8	0		2	6	0	
T4	10	5	5		8	1	1		8	2		4	5	1	
**Lymphe Node status**				0.89				**0.055**			0.53				0.21
N0	9	5	4		6	3	0		6	3		1	8	0	
N1	19	8	11		16	0	3		17	2		8	8	3	
N2	12	5	7		8	4	0		10	2		3	9	0	
N3	10	4	6		9	1	0		8	2		3	7	0	
**Metastasis**				0.84				0.11			0.51				0.35
M0	29	14	15		21	7	1		23	6		9	19	1	
M +	9	4	5		8	0	1		8	1		4	4	1	
Unknown	12	4	8		10	1	1		10	2		2	9	1	

Variable between groups presented in frequency tables evaluated by Chi-Square test. Two sided *p* values are considered statistically significant if < 0.05 and are indicated in bold.

## Discussion

It remains challenging to build knowledge about the dynamism of the inflammatory tumor microenvironment and to understand the behavior of the main intersecting mediators. A growing number of studies have shown that inflammation plays a critical role in tumorigenesis^23^. The inflammatory tumors microenvironment is characterized by the presence of host leukocytes both in the supporting stroma and in tumor areas^24^. These leukocytes are commonly called Tumor-infiltrating lymphocytes (TILs) which are essential for establishing an immune antitumor response but they may contribute to cancer growth and immunosuppression associated with malignancy^25,26^. TILs can be adaptive immune cells or innate immune cells such as cytotoxic cells particularly NK cells which we are interested in studying in our cancer research field. Moreover, it is clear that the inflammatory status involves several molecules such as immune checkpoints. These molecules participate mutually but differently in cellular response mediated by NK cells, but the profile of these molecules is not well understood. Members of the B7 family have been shown to be important participants in the TME designing notably B7-H1 (or PD-L1) molecule which was widely studied in breast cancer and others solid tumors^27^. Others are less studied such as B7-H6 molecule which appears to be of considerable importance. In this study, we have described the expression of B7-H6 and PD-L1both on cancer cell and TILs in women breast carcinoma. Our results show for the first time the expression of B7-H6 by TILs suitably within solid tumors. We have analyzed the widest part of the biopsy because we are convinced that TMAs tissue formats might not provide sufficient representation of the TME notably, immune infiltrating cells. Previous studies have shown only the expression of B7-H6 by cancer cells but no study have reported their expression by immune cell when analyzes are performed on TMA tissues^18,20,28^. So, one must be vigilant in certain technical aspects which can limit or even distort results, this point was raised previously by Sobral-Leite and colleagues^29^.

Our study is the first to investigate the concomitant profile of B7-H6 and PD-L1expression in breast carcinoma tissues by exploring their relationship first, and then their implication in innate immunity through their associations with NK cells status, and finally by looking for their combined prognostic value. Our results have shown that the two immune checkpoint molecules of interest participate differently in breast carcinoma physiopathology despite the strong association between them. Originally, our data have shown that the biomarker B7-H6 has a different clinical significance depending on its expression either by tumor cells or by infiltrating immune cells. In fact, statistical analysis demonstrated that high levels of B7-H6 in tumor cells are strongly correlated with Her2 expression, this result is in agreement with those of Sun and colleagues^21^. However, high B7-H6 + TILs are strongly associated with SBR grade, ER expression and lymph node invasion. Secondly, patients with high B7-H6 tumor expression had worse survival than those with low B7-H6 expression, this result is similar to data from other researchers in both ovarian cancer and breast cancer^18,21^. Nonetheless, patients with high TILs-B7-H6 expression displayed better survival. Regarding PD-L1biomarker expression, our data showed that its expression by tumor cells or immune stromal cells has the same clinical significance. However, we have noted the strong association of PD-L1BCC and TILs-PD-L1with SBR grade. In addition, patient survival curves according to these two biomarker statuses display the same trends with no differences between strong and weak expressions (Supplementary Fig. 3a–d). These data are consistent with the findings of several studies, while others have shown the opposite. This point has been reviewed by numerous experts who have justified the discrepancy in clinical significance of PD-L1 immune checkpoint among published reports^27^. Discrepancy can be explained by the use of several technical supports by different teams and the lack of a complete standardized platform. On the other hand, the great heterogeneity between studied populations may explain also the observed differences.

We explored the relationship between B7-H6 and PD-L1and their impact on NK cells recruitment. A strong association between the profiles of PD-L1in tumor or immune cells with B7-H6-TILs status was noticed. In addition, both TILs-B7-H6 and TILs-PD-L1 are highly associated with NK-TILs status. These results consolidate the concept of the mutual crosstalk between tumor cells and immune cells involving different checkpoint molecules that control not only T cell activity, but also NK cells function in a synergistic manner. Knowing that NK cells are associated with better disease outcomes even as minority population in TILs (Supplementary Fig. 4), these cells play an important role in front of the tumor burden. Additionally, survival tests were conducted on different combining groups based on the expression of B7-H6, the prominent biomarker of this study, with the other immunological parameters that had been selected to associate with (PD-L1 and NK-TILs statuses). The strong expression of B7-H6 by cancer cells probably contribute to the bad progression of the disease among all subgroups. Inversely, the high expression of B7-H6 by the immune cells among different subgroups might be related with a recovery process within the immune system involving NK cells and contributing to better disease outcomes. According to NK-TILs density in cancerous tissues, we have noted the best OS belong to the group of combination with high NK-TILs and TILs-B7-H6 together. Thus, the immune checkpoints B7-H6 and PD-L1 are linked together and certainly with other molecules in a way which modulates the inflammatory tumor microenvironment. We speculate a double role which can polarize the immune response either towards an effective antitumor response or the opposite by maintaining a chronic inflammatory state leading to immunosuppression. Already, different mechanisms damping the function of NK cells have been shown through B7 molecules action. However, little is known about the expression of these molecules that act differently depending on their localization whether expressed on the cellular membrane or sequestered in the cytoplasm or even secreted. These concepts justify the complexity in understanding the mechanisms controlling the tumor inflammatory microenvironment through B7 molecules. Our results suggest a protective role of B7-H6 and PD-L1 molecules when co-expressions are manifested on lymphocytes infiltrating tumors.These cells probably produce interleukins, such as INF-γ, which modulate NK cells function. Indeed, NK cells are considered to be players of innate immune defenses; but more recently, they have been recognized as immunoregulatory cells, secreting pro-inflammatory cytokines and several chemokines^30^. Thus, NK cells may recruit and may be recruited to inflammatory sites where they can co-localize with other immune cells, including professional presenting cells such as dendritic cells. NK cells can cooperate with them and modulate immune response. Besides the immune surveillance role, tumor neovascularization is highly controlled by cytokines in the microenvironment, which may have an immunosuppressive action resulting in an increase in angiogenic factors in tumor tissues. High expression of inflammatory cytokines in the tumor microenvironment seems to be the reason for the low therapeutic efficacy of current antiangiogenic drugs.

To better elucidate the immunomodulatory mechanism of B7 molecules in immune cell behaviors and consequent cytokines balancing, it is important to combine more precise analyses of their location and to conduct analyses allowing biomarkers co-revelation. Therefore, the immunotherapy targeting checkpoint molecules must be revised. The use of combined immune checkpoint inhibitors may result in a reduction of tumor burden and patient benefit in some cases. In other situations, of its inefficacy might be due to the double role of these two molecules. Targeting other molecules from the B7 family can be an important stake that deserves further investigation to decipher their contribution in innate immunity monitoring. Several molecules must be targeted simultaneously according to the patient characteristics. We speculate that a combinatorial therapeutic strategy based on immunotherapy targeting immune checkpoint molecules, antiangiogenic molecules and anti-inflammatory drug in a scalable and personalized way might improve disease outcome.

## Materials and methods

### Patients and tissues samples

Formalin-fixed, paraffin-embedded tissue samples were collected from 156 patients with primary invasive breast carcinomas who underwent surgical resection at the Department of Gynecology and Obstetrics of the HediChaker University Hospital (Sfax, Tunisia). All procedures performed in this study were in compliance with the ethical standards of the institutional and the national research committee of Habib Bourguiba University Hospital and with the 1964 Helsinki declaration and its later amendments or comparable ethical standards. All specimens were analyzed at the Department of Pathology of the Habib Bourguiba University Hospital (Sfax, Tunisia) and all tumor tissues were confirmed as the serous breast cancer by using haematoxylin and eosin (H&E) staining after surgical resection. Clinical*-*pathological data of patients were retrieved from the hospital's electronic records and available paper records. They included age, histological grade, histological type, molecular subtype, tumor size, lymph node status, distant metastasis, and lymphovascular invasion. All tumors were graded according to Elston Ellis modification of the Scarff–Bloom–Richardson (SBR) histological grading system^31^. The clinical stage was determined according to TNM (tumor, lymph node and metastasis) classification adopted by the International Union Against Cancer^32^. Outcomes data were collected from patient follow-ups at the Department of Medical Oncology of the Habib Bourguiba University Hospital (Sfax, Tunisia). Overall survival (OS) was defined as time in days from the beginning of the study observation period until death. If the death event did not occur, then the OS was noted as the total study observation period in days. Disease free survival (DFS) was defined from the date of the end of treatment until recurrences or last follow-up.

### Immunohistochemistry

Formalin-fixed, paraffin-embedded tissue samples were cut into 5-μm-thick section. Before immunostaining, haematoxylin and eosin-stained standard slides were reviewed by experimented pathologist (S.C) from each section of breast cancer tissues. A representative tumor region and the corresponding formalin-fixed paraffin-embedded tissue block were selected for use in the tissue array block containing six fragments of 7 mm in diameter.

In order to validate the immunohistochemical staining for special biomarker, striated muscle and placenta tissues were used as positive controls for the revelation of B7-H6 and PD-L1 respectively according to the instructions proposed by the manufacturers. For immunohistochemistry (IHC), each tumor array was cut into 2-μm sections and mounted on Leica Microsystems BOND Plus slides and dried overnight at 60 °C. Immunohistochemistry steps were performed as described in our previous study^33^. Briefly, tissue slides were deparaffinized in xylene followed by subsequent rehydration through graded alcohols then washed in purified water. Heat-induced antigen retrieval was performed at 95 °C for 20 min in in a pH = 9 epitope retrieval solution (Leica Novocastra) for 40 min. After heating, slides were allowed to cool down to room temperature and were briefly washed in phosphate-buffered saline (PBS) solution. Immunohistochemical staining was performed using the Novolink Polymer Detection System (RE7150-K, Leica Biosystems). Briefly, endogenous peroxidase activity was neutralized by Peroxidase Block for 5 min. Slides were washed with PBS, followed by application of Protein Block for 30 min. Following another PBS wash, tissue sections were incubated for 60 min at room temperature with primary antibody anti-PD-L1 at 1:100 (Human B7-H1, AF156 R&D systems), or anti-B7-H6 at 1 : 100 (Human B7-H6, NBP1-91,144 Novusbio) or with the anti-CD56 at 1:50 (NCL-L-CD56-1B6, Leica Novocastra). Slides were washed with PBS-Tween, then, only PD-L1 and CD56 antibodies reaction have been submitted to an adequate post primary antibody according to their species for 30 min. All tests received Novolink polymer solution for 30 min. DAB (Novolink DAB substrate) was used as the chromogen and Novolinkhematoxylin as the nuclear counterstain. Sections were dehydrated, cleared and mounted.

### Tumor immunostaining scoring and molecular subtyping

The B7-H6 immunostaining densities in tumor cells were analyzed according to the H-score method which has been described in a previous publication^34^. Briefly, H-score was assessed on the basis of the percentage of positive tumor cells with clearly brown cytoplasm and/or membrane immunostaining. H-score was calculated as follows = (% tumor cells unstained × 0) + (% tumor cells stained weak × 1) + (% tumor cells stained moderate × 2) + (% tumor cells stained strong × 3), and it ranged from 0 (100% negative tumor cells) to 300 (100% strong staining tumor cells). B7-H6 expression was dichotomized into two groups according to the frequency distributions of the H-scores displaying the median as the cut-off value (H-score 0–99 = negative/low expression, and 100–300 = positive/high expression). Frequency and staining intensity of PD-L1 by tumor cells were analyzed, and PD-L1 expression was quantified using the H-score method according to the previous publications^22,35^.

Breast cancer molecular classification is based on the expression of classical biomarkers including estrogen (ER) and progesterone (PR) receptors, the human epidermal growth factor receptor 2 (Her2) and Ki-67 labeling index as a cell proliferation biomarker. Expressions of all biomarkers were carried out using the immunohistochemical method. Five molecular subtypes were defined: Luminal A (LA) if ER/PR + , Her2- and Ki-67 < 20%; Luminal B like (LB-Like) if ER/PR + , Her2- and Ki-67 ≥ 20%; Luminal B (LB) if ER/PR + and Her2 + ; HER2 (H) if ER/PR- and HER2 + ; Triple Negative Breast Cancer (TNBC) if ER/PR- and Her2-^36,37^.

### Assessment of tumor-infiltrating lymphocytes (TILs)

Based on cellular morphological characteristics, TILs compartmentswere identified on cancerous tissues by haematoxylin staining at the end of the immunohistochemistry protocol. Subsequently, TILs-B7-H6 or TILs-PD-L1 were identified by the corresponding immunostaining through a brown staining on these cells. TILs-PD-L1 were evaluated in at least 3–4 representative high-power field (HPF) areas from each sample, and the mean PD-L1 + TILs count was scored using the following three-tier scoring system: 0–5%/HPF (Low), 5–50%/HPF (Medium), > 50%/HPF (High)^38^. For TILs-B7-H6 assessment, we applied the same strategy deployed in PD-L1enumeration due to the absence of recent publication showing TILs-B7-H6 expression in solid tumor, so we have considered the three-tier scoring described above. TILs CD56 + were assessed differently from TILs-PD-L1and TILs-B7-H6. TILs CD56 + analysis was performed as described in our previous study^33^. Briefly, the number of positive stromal and intratumoral CD56 + lymphocytes was quantified. Scoring of NK-TILs immunostaining was determined as positive or negative by a cut-off value of five cells in ten high power fields (× 40 magnification), yielding an immunoscore (NK-TILs) of Negative (< 5 cells) or Positive (≥ 5 cells).

### Statistical analysis

Statistical analyses were performed using the R language and the SPSS 20.0 statistical software for Windows (SPSS Inc., IBM). Bivariate analysis was performed to assess the correlation between biomarkers and clinical pathological characteristics using Chi-square tests for qualitative variables, Pearson-rank correlation for quantitative variables and ANOVA test for quantitative variation among classes of qualitative variables. The cumulative survival (overall survival, OS; recurrence-free survival, RFS) times were calculated using the Kaplan–Meier method and compared with the log-rank test. A cox-regression was also performed in order to evaluate the significance of prognostic factors on survival in a multivariate context (adjusting for confounding variables). All *p*-values less than 0.05 were considered significant.

### Ethical approval

All procedures performed in studies involving human participants were in accordance with the ethical standards of the institutional and the national research committee of Habib Bourguiba University Hospital and with the 1964 Helsinki declaration and its later amendments or comparable ethical standards. Sampling was made only on patient tissues from tissue library of Pathology Department-Habib Bourguiba Hospital and no samples were made specifically for the study.

## Supplementary Information

Supplementary Information

## Abbreviations

BC: Breast cancer
BCC: Breast cancer cell
TILs: Tumor infiltrating lymphocytes
NK: Natural killer
NK-TILs: Tumor infiltrating natural killer cell
TME: Tumor microenvironment
IHC: Immunohistochemistry
OS: Overall survival
DFS: Disease free survival
SBR: Scarff–Bloom–Richardson
TNM: Tumor, lymph node and metastases
LA: Luminal A
LB-Like: Luminal B like
H: Her2 positive
TNBC: Triple negative breast cancer
PBS: Phosphate-buffered saline

## References

[1] Jemal A (2011). Global cancer statistics. CA Cancer J. Clin..

[2] Dunn GP, Old LJ, Schreiber RD (2004). The immunobiology of cancer immunosurveillance and immunoediting. Immunity.

[3] Mittal D, Gubin MM, Schreiber RD, Smyth MJ (2014). New insights into cancer immunoediting and its three component phases–elimination, equilibrium and escape. Curr. Opin. Immunol..

[4] Moynihan KD, Irvine DJ (2017). Roles for innate immunity in combination immunotherapies. Cancer Res..

[5] Deniz G (2008). Regulatory NK cells suppress antigen-specific T cell responses. J. Immunol..

[6] Morandi B (2009). NK cells provide helper signal for CD8+ T cells by inducing the expression of membrane-bound IL-15 on DCs. Int. Immunol..

[7] Vitale M (2005). NK-dependent DC maturation is mediated by TNFalpha and IFNgamma released upon engagement of the NKp30 triggering receptor. Blood.

[8] Beldi-Ferchiou A, Caillat-Zucman S (2017). Control of NK cell activation by immune checkpoint molecules. Int J. Mol. Sci..

[9] Chen Q (2019). B7-H5/CD28H is a co-stimulatory pathway and correlates with improved prognosis in pancreatic ductal adenocarcinoma. Cancer Sci..

[10] Kasten BB, Ferrone S, Zinn KR, Buchsbaum DJ (2019). B7-H3-targeted radioimmunotherapy of human cancer. Curr. Med. Chem..

[11] Song X (2016). Prognostic role of high B7–H4 expression in patients with solid tumors: a meta-analysis. Oncotarget.

[12] Wang B, Ran Z, Liu M, Ou Y (2019). Prognostic significance of potential immune checkpoint member HHLA2 in human tumors: a comprehensive analysis. Front. Immunol..

[13] Flajnik MF, Tlapakova T, Criscitiello MF, Krylov V, Ohta Y (2012). Evolution of the B7 family: co-evolution of B7H6 and NKp30, identification of a new B7 family member, B7H7, and of B7’s historical relationship with the MHC. Immunogenetics.

[14] Brandt CS (2009). The B7 family member B7–H6 is a tumor cell ligand for the activating natural killer cell receptor NKp30 in humans. J. Exp. Med..

[15] Matta J (2013). Induction of B7–H6, a ligand for the natural killer cell-activating receptor NKp30, in inflammatory conditions. Blood.

[16] Wang L (2011). VISTA, a novel mouse Ig superfamily ligand that negatively regulates T cell responses. J. Exp. Med..

[17] Schlecker E (2014). Metalloprotease-mediated tumor cell shedding of B7-H6, the ligand of the natural killer cell-activating receptor NKp30. Cancer Res..

[18] Zhou Y (2015). B7-H6 expression correlates with cancer progression and patient’s survival in human ovarian cancer. Int. J. Clin. Exp. Pathol..

[19] Guo J-G (2016). Clinical significance of B7-H6 protein expression in astrocytoma. Onco Targets Ther.

[20] Zhou H (2019). The prognostic value of B7-H6 in esophageal squamous cell carcinoma. Sci. Rep..

[21] Sun J (2017). Clinical significance of novel costimulatory molecule B7-H6 in human breast cancer. Oncol. Lett..

[22] Li Z (2016). PD-L1 expression is associated with tumor FOXP3(+) regulatory T-cell infiltration of breast cancer and poor prognosis of patient. J Cancer.

[23] Karin M (2006). Nuclear factor-kappaB in cancer development and progression. Nature.

[24] Negus RP, Stamp GW, Hadley J, Balkwill FR (1997). Quantitative assessment of the leukocyte infiltrate in ovarian cancer and its relationship to the expression of C-C chemokines. Am. J. Pathol..

[25] Lin W-W, Karin M (2007). A cytokine-mediated link between innate immunity, inflammation, and cancer. J. Clin. Invest..

[26] Smyth MJ, Dunn GP, Schreiber RD (2006). Cancer immunosurveillance and immunoediting: the roles of immunity in suppressing tumor development and shaping tumor immunogenicity. Adv. Immunol..

[27] Matikas A (2019). Prognostic implications of PD-L1 expression in breast cancer: systematic review and meta-analysis of immunohistochemistry and pooled analysis of transcriptomic data. Clin. Cancer Res..

[28] Jiang T (2017). High expression of B7-H6 in human glioma tissues promotes tumor progression. Oncotarget.

[29] Sobral-Leite M (2018). Assessment of PD-L1 expression across breast cancer molecular subtypes, in relation to mutation rate, BRCA1-like status, tumor-infiltrating immune cells and survival. Oncoimmunology.

[30] Melaiu O, Lucarini V, Cifaldi L, Fruci D (2020). Influence of the tumor microenvironment on NK cell function in solid tumors. Front. Immunol..

[31] Elston CW, Ellis IO (2002). Pathological prognostic factors in breast cancer. I. The value of histological grade in breast cancer: experience from a large study with long-term follow-up. Histopathology.

[32] O’Sullivan B (2017). The TNM classification of malignant tumours-towards common understanding and reasonable expectations. Lancet Oncol..

[33] Triki H (2019). CD155 expression in human breast cancer: clinical significance and relevance to natural killer cell infiltration. Life Sci..

[34] Pesce S (2015). B7-H6-mediated downregulation of NKp30 in NK cells contributes to ovarian carcinoma immune escape. Oncoimmunology.

[35] Muenst S (2014). Expression of programmed death ligand 1 (PD-L1) is associated with poor prognosis in human breast cancer. Breast Cancer Res. Treat..

[36] Hammond MEH, Hayes DF, Wolff AC, Mangu PB, Temin S (2010). American society of clinical oncology/college of american pathologists guideline recommendations for immunohistochemical testing of estrogen and progesterone receptors in breast cancer. J. Oncol. Pract..

[37] Wolff AC (2018). Human epidermal growth factor receptor 2 testing in breast cancer: american society of clinical oncology/College of American Pathologists clinical practice guideline focused update. J. Clin. Oncol..

[38] Salgado R (2015). The evaluation of tumor-infiltrating lymphocytes (TILs) in breast cancer: recommendations by an International TILs Working Group 2014. Ann. Oncol..

