# Supplementary material for: Immune checkpoint molecules B7-H6 and PD-L1 co-pattern the tumor inflammatory microenvironment in human breast cancer
Source: Sci Rep. 2021 Apr 6;11:7550. doi: 10.1038/s41598-021-87216-9 (PMC8024320; doi:10.1038/s41598-021-87216-9)
Supplement: Supplementary file 1 — Supplementary Information [file 41598_2021_87216_MOESM1_ESM.docx]

# TITLE

# Immune checkpoint molecules B7-H6 and PD-L1 co-pattern the tumor inflammatory microenvironment in human breast cancer

**AUTHORS**

Boutheina Cherif^1*^,Hana Triki^1,2^, Slim Charfi^2^,Lobna Bouzidi^2^, [Wala Ben Kridis](https://www.ncbi.nlm.nih.gov/pubmed/?term=Kridis%20WB%5BAuthor%5D&cauthor=true&cauthor_uid=26628769)^3^, Afef Khanfir^3^, Kais Chaabane^4^,Tahya Sellami-Boudawara^2^, and Ahmed Rebai^1^

**Authors’ affiliation**

^1^Laboratory of Molecular and Cellular Screening Processes, Center of Biotechnology of Sfax, Sfax University, Sfax, Tunisia

^2^Department of Pathology, University Hospital Habib Bourguiba, Sfax, Tunisia

^3^Department of Medical Oncology, University Hospital Habib Bourguiba, Sfax, Tunisia

^4^Department of Gynecology, University Hospital Hédi Chaker, Sfax, Tunisia

***Corresponding author**

E-mail: [boutheina.cherif.cbs@gmail.com](mailto:boutheina.cherif.cbs@gmail.com)

[boutheina.cherif@cbs.rnrt.tn](mailto:boutheina.cherif@cbs.rnrt.tn)

ORCID: <https://orcid.org/0000-0002-6923-2948>

Personal Phone: (+ 216) 99 825 555

Postal Address: Center of Biotechnology of Sfax, B.P 1177 Sfax 3018 Tunisia

Institutional Phone: (+ 216) 74 871 816 / 74 875 817

Institutional Fax: (+ 216) 74 87 58 18

Institutional website: <http://www.cbs.rnrt.tn/>

**Supplementary Table: Associations of B7-H6 expression with PD-L1 and NK-TILs statutes in breast cancer tissues**

| **ImmueParameter** | All patients n = 156 | **B7-H6 BCC** | | *P*-value | **TILs-B7-H6** | | | *P*-value | **PD-L1 BCC** | | | *P*-value | **TILs-PD-L1** | | | *P*-value |
| --- | --- | --- | --- | --- | --- | --- | --- | --- | --- | --- | --- | --- | --- | --- | --- | --- |
|  |  | <median n=96 | ≥ median n=60 |  | Low n = 126 | High n = 20 | Unknown n = 10 |  | <median n=125 | ≥ median n=31 | |  | Low n = 58 | High n = 88 | Unknown n = 10 |  |
| **TILs-B7-H6** |  |  |  | 0.91 |  |  |  |  |  |  |  | |  |  |  |  |
| Low | 126 | 81 | 45 |  |  |  |  |  |  |  |  | |  |  |  |  |
| High | 20 | 6 | 14 |  |  |  |  |  |  |  |  | |  |  |  |  |
| Unknown | 10 | 9 | 1 |  |  |  |  |  |  |  |  | |  |  |  |  |
| **PD-L1 BCC** |  |  |  | 0.57 |  |  |  | **<0,01**** |  |  |  | |  |  |  |  |
| Low | 125 | 77 | 48 |  | 106 | 13 | 6 |  |  |  |  | |  |  |  |  |
| High | 31 | 19 | 12 |  | 20 | 7 | 4 |  |  |  |  | |  |  |  |  |
| **TILs-PD-L1** |  |  |  | 0.088 |  |  |  | **0.026*** |  |  | 0.28 | |  |  |  |  |
| Low | 58 | 40 | 18 |  | 58 | 0 |  |  | 50 | 8 |  | |  |  |  |  |
| High | 88 | 47 | 31 |  | 68 | 20 |  |  | 69 | 19 |  | |  |  |  |  |
| Unknown | 10 | 9 | 1 |  |  |  | 10 |  | 6 | 4 |  | |  |  |  |  |
| **Nk-TILs** |  |  |  | 0.35 |  |  |  | **0.041*** |  |  | **0.023*** | |  |  |  | **0.028*** |
| Low | 111 | 69 | 42 |  | 100 | 11 |  |  | 95 | 16 |  | | 50 | 61 |  |  |
| High | 35 | 18 | 17 |  | 26 | 9 |  |  | 24 | 11 |  | | 8 | 27 |  |  |
| Unknown | 10 | 9 | 1 |  |  |  | 10 |  | 6 | 4 |  | |  |  | 10 |  |

Variable between groups presented in frequency tables evaluated by Chi-Square test. Two sided p-values are considered statistically significant if <0.05 and are indicated in bold.

**Supplementary figure 1. B7-H6 and PD-L1 H-scores associations with TILs-PD-L1, TILs-B7-H6 and NK-TILs statutes in Her2 positive cancer subtype.**

Boxplot representations of H-score PD-L1 distribution according to TILs-PD-L1 status (**a**, *p* = 0.0293), TILs-B7-H6 status (**b**, *p* = 0.0174) and NK-TILs status (**c**, *p* = 0.0293). Boxplot representations of H-score B7-H6 distribution according to TILs-PD-L1 status (**d**, *p* = 0.137), TILs-B7-H6 status (**e**, *p* =0.051) and NK-TILs status (**f**, *p* = 0.074).

**
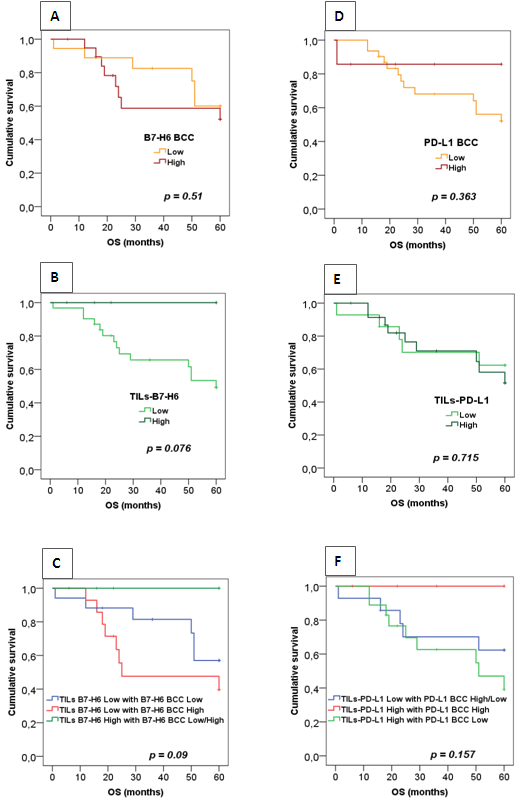
**

**Supplementary figure 2. Survival analysis according to B7-H6 and PD-L1 expression in Her2 positive breast cancer tissues.**

(**a** and **d**) Kaplan-Meier curves for OS stratified according to B7-H6 and PD-L1 expression by cancer cells (B7-H6 BCC) and (PD-L1 BCC) respectively. (**b** and **e**) Kaplan-Meier curves for OS stratified according to B7-H6 and PD-L1 expression by immune infiltrating cells (TILs-B7-H6) and (TILs-PD-L1) respectively. (**c**) OS curves according to B7-H6 expression by tumor and immune cells together leading to three subgroups; subgroup 1: TILs-B7-H6^Low^/ B7-H6 BCC^Low^, subgroup 2: TILs-B7-H6^Low^/ B7-H6 BCC^High^ and subgroup 3: TILs-B7-H6^High^/ B7-H6 BCC^Low/High^. (**f**) OS curves according to PD-L1 expression by tumor and immune cells together leading to three subgroups; subgroup 1: TILs-PD-L1^Low^/ PD-L1 BCC^High/Low^, subgroup 2: TILs- PD-L1^High^/ PD-L1 BCC^High^ and subgroup 3: TILs- PD-L1^High^/ PD-L1 BCC^Low^.


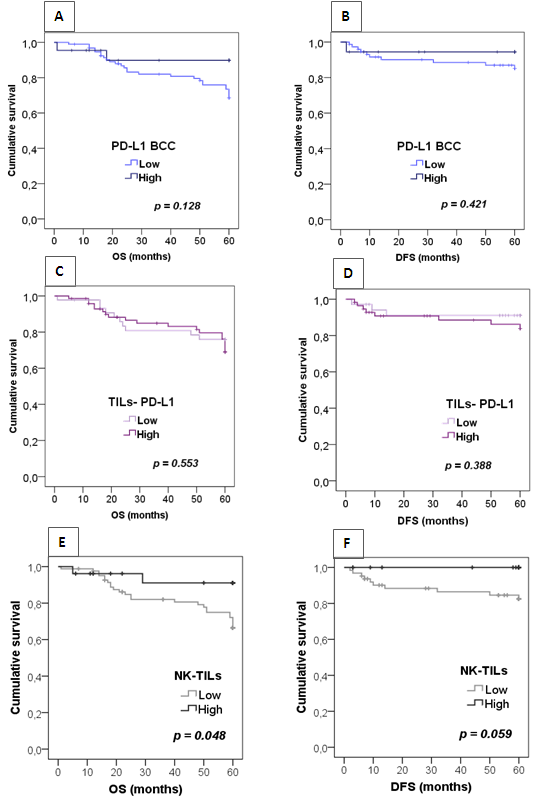


**Supplementary figure 3. Survival analysis according to PD-L1 expression and NK-TILs status in breast cancer tissues.**

(**a**-**b**) Kaplan-Meier curves stratified according to PD-L1 expression by cancer cells (PD-L1 BCC) for overall survival (OS) and disease free survival (DFS). (**c-d**) Kaplan-Meier curves stratified according to PD-L1 expression by immune infiltrating cells (TILs-PD-L1) for OS and DFS. (**e-f**) Kaplan-Meier curves stratified according to NK cell infiltration (NK-TILs) for OS and DFS.


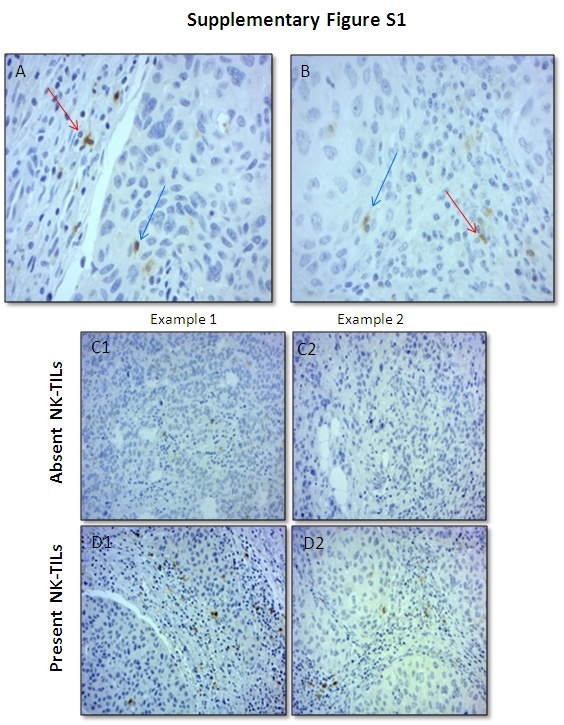


**Supplementary figure 4. Immunohistochemical evaluation of CD56+ NK-TILs in breast cancer**.

(**a-b**) Histological section showing CD56+NK cells localizations for intratumoral (blue arrows) and stromal (red arrows) areas. Representative images for absent (**c1-c2**) and present (**d1-d2**) CD56+NK cells. (Original magnification ×400)
